# Egg turning behavior and incubation temperature in Forster’s terns in relation to mercury contamination

**DOI:** 10.1371/journal.pone.0191390

**Published:** 2018-02-15

**Authors:** Gregory T. Taylor, Joshua T. Ackerman, Scott A. Shaffer

**Affiliations:** 1 Department of Biological Sciences, San Jose State University, San Jose, California, United States of America; 2 U.S. Geological Survey, Western Ecological Research Center, Dixon Field Station, California, United States of America; CEFE, FRANCE

## Abstract

Egg turning behavior is an important determinant of egg hatchability, but it remains relatively understudied. Here, we examined egg turning rates and egg temperatures in Forster’s terns (*Sterna forsteri*). We used artificial eggs containing a data logger with a 3-D accelerometer, a magnetometer, and a temperature thermistor to monitor parental incubation behavior of 131 tern nests. Overall, adults turned their eggs an average (±SD) of 3.8 ± 0.8 turns h^-1^, which is nearly two times higher than that of other seabirds. Egg turning rates increased with nest initiation date. We also examined egg turning rates and egg temperatures in relation to egg mercury contamination. Mercury contamination has been shown to be associated with reduced egg hatchability, and we hypothesized that mercury may decrease egg hatchability via altered egg turning behavior by parents. Despite the high variability in egg turning rates among individuals, the rate of egg turning was not related to mercury concentrations in sibling eggs. These findings highlight the need for further study concerning the potential determinants of egg turning behavior.

## Introduction

Avian incubation involves a variety of parental behaviors that can affect the hatching success of eggs. One behavior in particular, egg turning, plays a key role in ensuring proper embryonic development [1–5]. Egg turning facilitates the absorption of the albumen by the embryo [6] and prevents the embryo from adhering to the shell membrane [7,8]. Studies on domestic fowl have shown that failure to turn eggs can lower hatching rates by as much as 50% [9].

Environmental contaminants like mercury can also influence egg hatchability [10–12]. However, the mechanism by which mercury impairs egg hatching success is unknown. Previous research suggested that mercury may increase the incidence of embryo malpositions [12], possibly through altered parental care, which can lead to reduced egg hatchability [4,13–15]. Mercury is a known endocrine disruptor that interacts with prolactin [16], testosterone [17], and corticosterone [17–20], all of which are strongly associated with incubation behavior [21–24]. Although mercury has been shown to alter incubation behaviors (such as nest attendance) in a number of avian species [25], specific behaviors like egg turning rates or patterns have not been examined simultaneously with measures of mercury contamination. Egg temperatures also may be affected by the egg turning behavior of parents [26] and by altered parental nest attendance. Thus, mercury contamination may lead to suboptimal egg turning rates [1,4,14,27] and abnormal egg temperatures [13,15,28,29] that could result in the malpositioning of an embryo or other impairment that could reduce egg hatchability.

To improve our knowledge of incubation behavior, particularly egg turning rates, we studied Forster’s terns (*Sterna forsteri*) nesting in San Francisco Bay, California. Forster’s terns have the highest mean mercury concentrations of any bird in San Francisco Bay [30,31] and western North America [25]. To investigate the relationship between mercury contamination and incubation behavior, egg mercury concentrations were used as a proxy for parental mercury concentrations because previous research demonstrated that mercury in Forster’s tern eggs is strongly correlated with mercury in the incubating parents (*R*^2^ = 0.95 between mother and egg, and *R*^2^ = 0.84 between father and egg) [32]. Additionally, we examined the relationship between egg turning rate and egg temperature, and compared the egg turning behavior of Forster’s terns with other birds.

We used multivariate regression analyses to evaluate the influence of a suite of variables, including nest initiation date, incubation stage, clutch size, day vs. night (a categorical), and egg mercury concentration, as potential predictors of egg turning rate and egg temperature. We assumed that a higher egg turning rate was indicative of increased parental attentiveness. Therefore, we predicted that (1) egg turning rate would decrease with nest initiation date, because lower-quality [33] and less-experienced [34] terns tend to nest later in the breeding season and therefore may be less attentive; (2) egg turning rate would increase with incubation stage, since adults typically become more attentive as the incubation stage progresses [35,36]; (3) egg turning rate would decrease with clutch size, because a larger number of eggs in the nest may inhibit the movement of individual eggs; (4) egg turning rate would increase during the day and decrease at night, because adult Forster’s terns are more active (e.g., foraging, mobbing predators, exchanging incubation duties) during the day than at night [37,38]; and (5) egg turning rate would decrease with mercury concentration, because mercury has been linked to reduced nest attendance [39] and egg neglect [16]. In regards to egg temperature, we predicted that (1) egg temperature would increase with nest initiation date, since ambient temperatures increase over the course of the breeding season at our study site; (2) egg temperature would increase with incubation stage, because parents generally become more attentive as the incubation stage progresses [35,36]; (3) egg temperature would not vary with clutch size, because the maximum clutch size of three eggs matches the number of brood patches on the adults [40]; (4) egg temperature would increase during the day and decrease at night, following the circadian rhythm of the parents’ body temperatures [41,42,43]; (5) egg temperature would decrease with mercury concentration, because mercury has been associated with reduced nest attendance [39] and egg neglect [16]; and (6) egg temperature would increase with egg turning rate, because a higher egg turning rate may be indicative of greater parental attendance.

## Methods and materials

### Study species and field site

The San Francisco Bay, recognized as a wetland of international significance [44] and a site of hemispheric importance for shorebirds [45], is a key stopover site along the Pacific Flyway [46] and serves as overwintering habitat for nearly 1 million waterbirds each year [47]. The Forster’s tern is a piscivorous seabird that forages in the marshes and salt ponds along the perimeter of the Bay [48], where mercury levels are particularly high [49].

The incubation behavior and mercury contamination levels of Forster’s terns (hereafter “terns”) were studied at five colonies in the south San Francisco Bay, CA at the Don Edwards National Wildlife Refuge (Ponds AB1, AB2, A2W, R1, and New Chicago Marsh; 37°26’N, 122°3’W) from April-July 2015.

### Egg collection for mercury evaluation

We randomly collected one tern egg for mercury determination and replaced it with an artificial egg. Eggs were only collected from nests that contained at least two eggs and only if all eggs in the nest were 6–12 days into the 24-day incubation period. Incubation stage was determined by egg floatation [50,51]. Collected eggs were stored in a refrigerator at 4°C. All eggs were collected under a California Department of Fish and Wildlife Permit (SC-13121), a United States Fish and Wildlife Service Migratory Bird Permit (MB173904-4), and the San Jose State University Institutional Animal Care and Use Committee (SJSU 1022).

### Artificial egg logger deployment

After removing an egg for mercury assessment, an artificial egg containing a data logger (see [52] for details) with an onboard temperature thermistor, triaxial accelerometer and magnetometer, and a removable 4 GB microSD flash memory card was placed in the nest. At 1 second intervals, egg loggers recorded temperature (resolution 0.125°C, < 2°C accuracy) and 3-D orientation (resolution 1–2°).

All artificial eggs used for logger deployments were made by Advanced Assembly (Aurora, CO) on a 3-D printer. Eggs were sized (45 mm length x 30 mm maximum breadth) to match the shape of Forster’s tern eggs [53]. Eggs also were filled with barium sulfate (BaSO_4_), a non-toxic non-ferrous powder that does not affect the functioning of the sensors, to ensure the mass of the artificial eggs approximated that of a Forster’s tern egg (21.1 ± 1.6 g SD; [53]). These methods ensured that artificial egg temperatures approximated those of real eggs. Each logger was positioned along the long axis of the egg in the center of each artificial egg, so all temperature measurements are considered core egg temperature. Printed eggs were then painted to match the color and pattern of tern eggs, using non-toxic water-based paint.

Potential biases due to changes in incubation behavior over the course of the incubation period [5,54,55] were reduced by deploying egg loggers in nests that were at 6–12 days into the incubation cycle. Incubation stage also was included as a covariate in our statistical models (described below). Egg loggers were left in each nest for one week, but data were recorded for a mean of 5.8 ± 1.2 SD days.

### Egg dissection and processing

The length and breadth of each egg was measured to the nearest 0.01 mm using digital calipers (Fowler, Newton, MA), then weighed to the nearest 0.01 g using a digital balance (Ohaus Adventurer Pro, Ohaus Corporation, Pine Brook, NJ). A hole approximately 15-mm in diameter was cut in the top of each egg using clean, stainless steel scissors. Egg contents were transferred to 30 mL vials using stainless steel forceps, then weighed with a digital scale to the nearest 0.01 g. Egg contents were then frozen at -20°C until further analysis. Thawed egg contents were subsequently dried at 50°C for 48 h or until completely dried, then reweighed to determine each egg’s percent water content. Using a mortar and pestle, egg contents were homogenized to a powder and thereafter stored in a desiccator until mercury content determination.

### Mercury determination

Mercury analysis was conducted at the U.S. Geological Survey, Dixon Field Station Environmental Mercury Laboratory. Total mercury was used as an index of methyl mercury because 96% of the total mercury in bird eggs is in the methyl mercury form [56]. Total mercury concentrations were determined on a Nippon MA-3000 Direct Mercury Analyzer (Nippon Instruments, College Station, Texas) following Environmental Protection Agency Method 7473 [57]. Total mercury (THg) concentrations in eggs were determined on a dry weight basis and then converted into a fresh wet weight egg concentration using egg-specific water content and egg morphometrics following the methods of Ackerman et al. [56] and egg densities specific to this species [53].

Quality assurance measures included analyses of two certified reference materials (either dogfish muscle tissue [DORM], dogfish liver [DOLT], or lobster hepatopancreas [TORT] certified by the National Research Council of Canada, Ottawa, Canada, or fish homogenate [IAEA] certified by the International Atomic Energy Agency), two system and method blanks, three continuing calibration verifications, and two duplicates per batch. Recoveries (mean ± SE) were 100% ± 0.9% (n = 45) for certified reference materials, and 99.8% ± 0.2% (n = 44) for continuing calibration verifications. Absolute relative percent difference for duplicates averaged 2.8 ± 0.4% (n = 38).

### Data processing

All data analyses were conducted using custom routines created in MATLAB (The MathWorks, Natick, MA). Following methods of Shaffer et al. [52], raw accelerometer and magnetometer measurements were converted to 3-2-1 Euler angles (yaw, pitch, and roll), to estimate instantaneous egg orientation. Yaw orientation refers to side-to-side movements of the egg, pitch refers to up-and-down movements, and roll refers to the rotation of the egg around the long axis. To account for minor postural changes of a bird sitting on its eggs, we only considered total orientation changes greater than 10° as an egg turning event. This threshold also has been used in similar studies [52,54,58], and it approximates the inflection point in the cumulative distribution between angle change and egg turning rate.

Because our presence in the colony to monitor nests and conduct artificial egg deployments disrupted normal incubation behaviors, all data within 2.5 h of egg logger deployment or logger recovery were excluded from analysis.

Hourly and daily egg turning rates, as well as mean hourly and daily temperatures, were determined for each egg logger deployment, consistent with previous studies [26,52,54]. Daily egg turning rate was characterized as the number of turns between consecutive sunrises. Sunrise and sunset times were determined from ephemeris tables using the latitude and longitude of colony A2W, which is centrally located among all the tern colonies.

### Statistical analyses

All egg mercury concentrations were natural log-transformed to improve normality, and are reported as micrograms per gram fresh wet weight (μg/g fww). Paired t-tests were used to determine whether hourly egg turning rate differed between day and night. We used three global multivariate regression models to 1) determine which explanatory variables were the most important predictors of hourly egg turning rate, 2) determine which explanatory variables were the most important predictors of hourly egg temperature, and 3) determine whether egg turning rate was an important predictor of egg temperature. Parameter estimation was conducted using Standard Least Squares. In all models, nest identification and colony were included as random effects to statistically nest data collected from the same nest or same colony.

In the first two models, which sought to determine the most important predictors of egg turning rate and egg temperature, the following explanatory variables were included as fixed effects: egg THg concentration, incubation stage, standardized nest initiation date, clutch size, and day vs. night (categorical). In the third model, which sought to determine whether egg turning rate was an important predictor of egg temperature, egg THg concentration was replaced with egg turning rate. Variance inflation factor was used to test for collinearity among the explanatory variables. Incubation stage was defined as the day of the incubation period (1–24 for Forster’s terns). We back-calculated the initiation date (accurate to within 3 days [51]) for each nest based on the incubation stage and number of eggs in the nest when the nest was first found in the colony, with incubation stage being an average of all eggs in the nest. Median nest initiation date for the species was determined and this was subtracted from each nest’s initiation date to standardize nest initiation date. Clutch size was defined as the total number of eggs in a nest during egg logger deployment, and includes the egg logger which replaced a real egg in the nest. A given hour was categorized as “day” if ≥50% of that hour occurred between sunrise and sunset; otherwise it was categorized as “night.”

All statistical analyses were conducted using JMP (SAS Institute, Cary, NC), using a significance level of p ≤ 0.05. All data are presented as mean ± 1 SD unless otherwise stated.

## Results

### Egg mercury levels

THg concentration in tern eggs was 1.47 ± 0.76 μg/g fww. These egg THg concentrations are similar to what has been found previously in this population [32] and, using equations developed previously for this species [32], are approximately equivalent to 3.19 μg/g wet wt in the incubating mother’s blood and 3.86 μg/g wet wt in the father’s blood.

### Factors related to egg turning rate

Of the six explanatory variables included in the multivariate regression, three were related to hourly egg turning rate in Forster’s terns. Hourly egg turning rate was negatively related to clutch size (Table 1). Hourly egg turning rate was also related to day vs. night. Hourly egg turning rate was not related to egg THg concentrations or incubation stage. Hourly egg turning rate was positively related to standardized nest initiation day. A variance inflation factor below 5 indicated that there was no collinearity among the explanatory variables.

**Table 1:** Statistical results of the three multiple regressions models.

Response Variable	Explanatory Variables					
	Nest Initiation Day	Clutch Size	Day vs. Night	Incubation Stage	THg	Egg Turning Rate
						
Egg Turning Rate	*F*_1,115.8_ = 33.95, *p* < 0.001	*F*_1,111.7_ = 4.64, *p* = 0.03	*F*_1,16481_ = 9.41, *p* < 0.001	*F*_1,5336_ = 0.008, *p* = 0.76	*F*_1,130.4_ = 0.07, *p* = 0.78	
Egg Temperature	*F*_1,122.4_ = 19.19, *p* < 0.001	*F*_1,111.9_ = 0.01, *p* = 0.92	*F*_1,16456_ = 10.93, *p* < 0.001	*F*_1,15809_ = 186.45, *p* < 0.001	*F*_1,270.9_ = 3.19, *p* = 0.08	
Egg Temperature	*F*_1,122.4_ = 19.19, *p* < 0.001	*F*_1,111.9_ = 0.01, *p* = 0.92	*F*_1,16456_ = 10.93, *p* < 0.001	*F*_1,15809_ = 186.45, *p* < 0.001	*F*_1,270.9_ = 3.19, *p* = 0.08	*F*_1,16759_ = 189.80, *p* < 0.001

Mean hourly egg turning rate was 3.8 ± 0.8 turns h^-1^. Hourly egg turning rates were more variable during the day, and also were significantly higher during the day (4.5 ± 1.1 turns h^-1^) than at night (2.8 ± 0.9 turns h^-1^).

**Fig 1:**
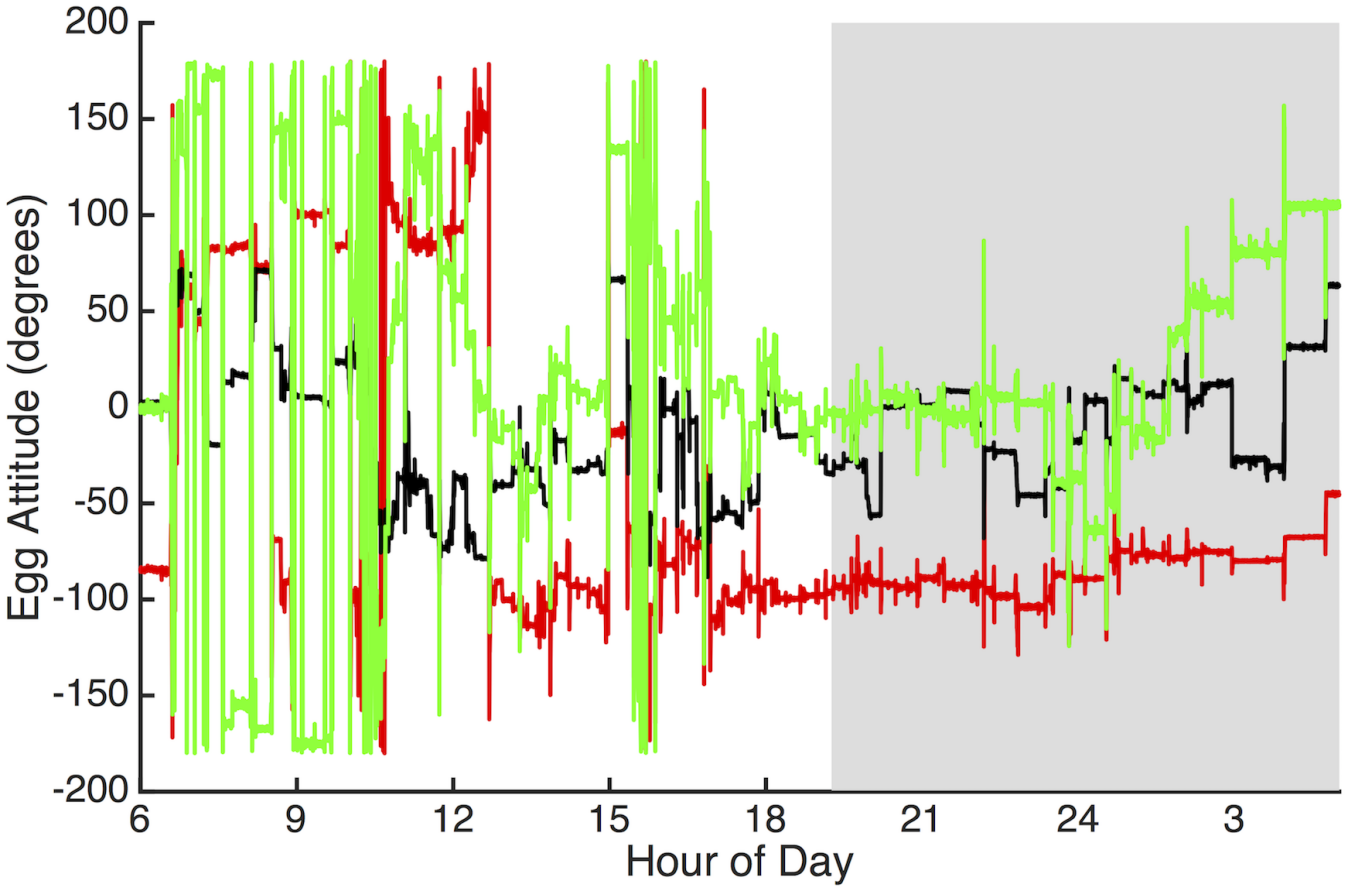
The three aspects of egg orientation (roll, pitch, and yaw) in a single Forster’s tern egg. White areas indicate daytime, while gray areas indicate nighttime. The red line represents roll, the black line indicates pitch, and the green line indicates yaw. Note the large change in yaw angle relative to roll and pitch, as well as the difference in egg turning activity between night and day.

In all tern nests, the axis of orientation that showed the greatest amount of change in each egg turning event was the yaw, followed by the roll, then the pitch (Fig 1). For each egg turning event, the mean angle-change was 59.0 ± 1.2 deg for the yaw attitude, 39.3 ± 0.6 deg for the roll, and 23.4 ± 0.3 deg for the pitch. The overall mean angle-change per egg turning event was 46.3 ± 0.5 deg.

### Factors related to egg temperature

Of the six explanatory variables included in the multivariate regression model, three were related to hourly egg temperature in Forster’s terns. Hourly egg temperature was positively related to incubation stage and standardized nest initiation day. Hourly egg temperature was also significantly related to day vs. night. There was no significant relationship between hourly egg temperature and egg THg concentration or clutch size. A variance inflaction factor below 5 indicated an absence of collinearity among explanatory variables.

Mean daytime egg temperature was 40.0 ± 2.5 °C and differed from mean nighttime egg temperature, which was 38.9 ± 2.4 °C. Hourly daytime egg temperatures also were more variable than hourly nighttime egg temperatures.

### Does egg turning rate influence egg temperature?

Of the six explanatory variables included in this model, hourly egg temperature was positively related to hourly egg turning rate, incubation stage, and standardized nest initiation day. Hourly egg temperature was also significantly correlated with day vs. night. Hourly egg temperature was not related to clutch size. No collinearity was detected among the explanatory variables (variance inflation factor < 5).

## Discussion

To our knowledge, the present study is the first to examine egg turning rates in terns. Overall, we found that Forster’s terns had a mean egg turning rate of 3.8 ± 0.8 turns h^-1^, which is nearly twice the rate for most other seabird species that have been studied. The mean angle change per egg turning event also was higher than what has been reported for other seabirds. We observed that egg turning rate increases with standardized nest initiation date while accounting for incubation stage. Additionally, Forster’s tern egg temperature was positively correlated with standardized nest initiation date and egg turning rate. Egg temperature range in Forster’s terns was higher than what has been found in most seabirds.

### Egg turning rate in Forster’s terns

Most species studied using similar technology average approximately 2 turns h^-1^ [26,52,55], which is typical of birds with semi-precocial offspring [5]. Egg turning rates have been reported for several gull species [59], but these studies included potential sources of inaccuracy (human disturbance, sampling only during the day, reliance on visual observations, small sample size, etc.) and only western gulls (*Larus occidentalis*) have been studied in detail (2.1 ± 0.4 turns h^-1^; [52,55]). Egg turning rates of Adélie penguins (*Pygoscelis adeliae*) varied from 1.4 to 3.2 times per hour [54,58,60], though those loggers only had a bi-axial accelerometer and lacked a magnetometer. Incorporating a magnetometer has been shown to record 10–30% more turning events [52]; thus, the estimates of egg turning rates of Adélie penguins are likely biased low. Therefore, egg turning rates in Forster’s terns appear to be higher than in other species measured thus far. Given that egg turning rate may vary among populations for any number of reasons (e.g., weather conditions [60] or disturbance levels), future investigations might examine egg turning rates in other Forster’s tern populations throughout their range.

One characteristic that differentiates all previous species from Forster’s terns, and that may explain the difference in turning rates, is the duration of their foraging bouts. Egg turning rate appears to be associated with foraging bout frequency in a number of species (discussed below), potentially due to the movement of the parents as they resettle on the nest, as observed in black-headed gulls (*Chroicocephalus ridibundus*) [61] and herring gulls (*Larus argentatus*) [35]. Forster’s terns at our study colonies generally forage within 3 km of their nests during the incubation period [62], and they commonly conduct foraging excursions that are less than 4 hours [63]. Consequently, parents exchange incubation duties multiple times over the course of a day. In comparison to Forster’s terns, western gulls, which turn their eggs 2.1 turns h^-1^, spend 2–4 hours away from the nest per foraging trip during the incubation period [64]. Cassin’s auklets (*Ptychoramphus aleuticus*), which turn their eggs 2.2 turns h^-1^, exchange incubation duties on a nightly basis (i.e., ca. 24 hours), where parents alternate between foraging at sea at night or sitting on the nest and incubating the egg during the day [26]. Both Adélie penguins (1.4–3.2 turns h^-1^) and Laysan albatrosses (*Phoebastria immutabilis*; 2.1 turns h^-1^) alternate prolonged incubation bouts with long foraging trips so parents exchange nesting duties less frequently [52,65]. Hence the high rate of egg turning in Forster’s terns may be explained by more frequent incubation bout exchanges between parents in comparison to other seabird species.

A second behavioral aspect that is common in terns is their propensity to mob predators [66]. Similar to the effects of frequent turnover between parents of the same nest, mobbing behavior in Forster’s terns could also increase the number of egg movements when adults flush off and resettle back on the nest. Predators, particularly California gulls (*Larus californicus*), are common at our colony sites and gulls account for 54% of Forster’s tern chick deaths [67].

**Fig 2:**
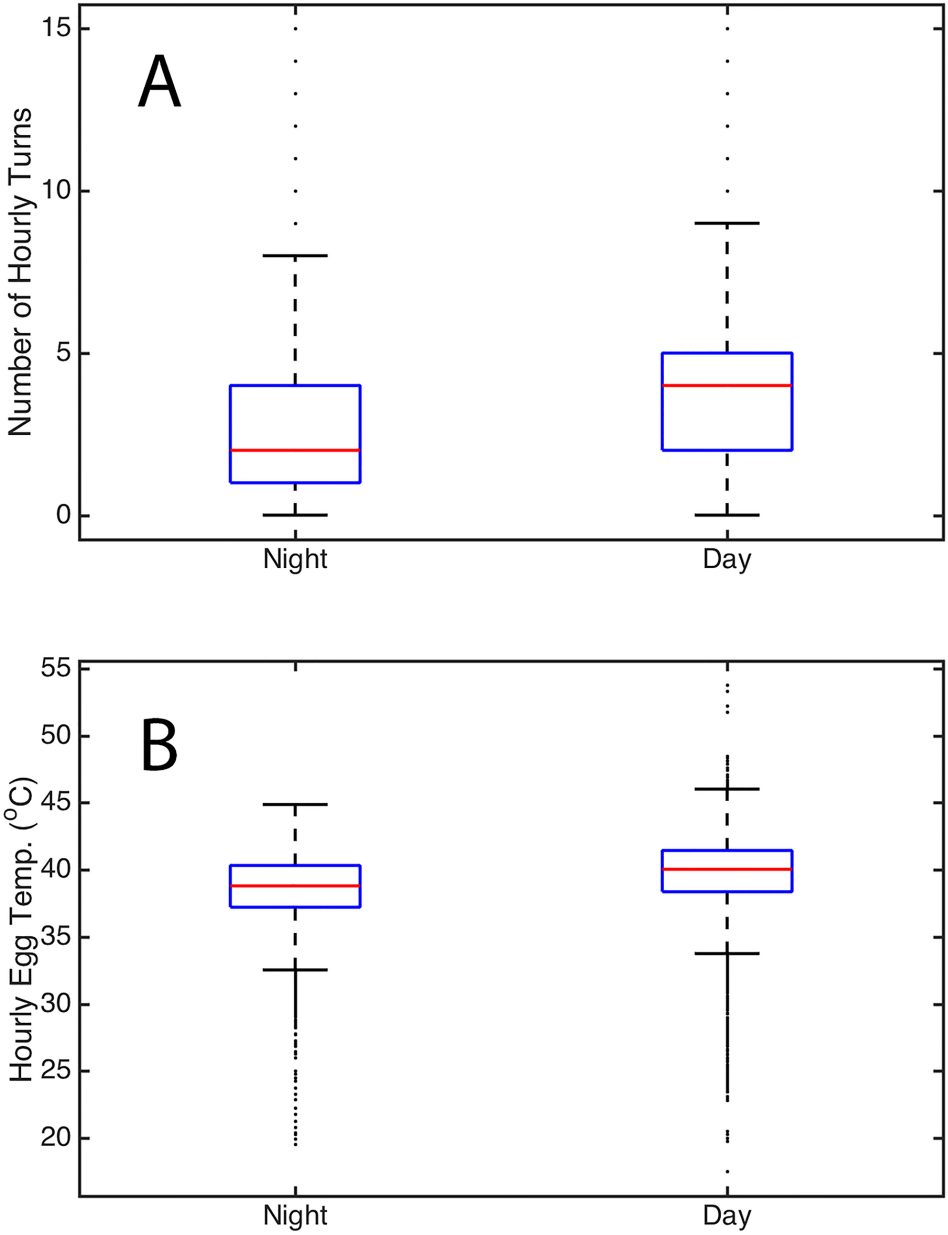
The hourly number of turns and egg temperature during the night and during the day in terns. (A) Hourly number of turns. (B) Egg temperature. The boxes denote the median, 25 & 75% quartiles, the range of data, and outliers.

Hourly egg turning rates in Forster’s terns were 60% higher during the day (4.5 ± 1.1 turns h^-1^) than at night (2.8 ± 0.9 turns h^-1^, Fig 2A). The higher daytime turning rate may be explained by the timing of the parents’ exchange of incubation duties, which are more frequent during the day [37]. Similarly, western gulls exchange incubation duties more often during the day [64], and exhibit increased daytime egg turning rates [55]. This pattern is also evident, but reversed, in the nocturnal Cassin’s auklet where egg turning rates are higher at night, when the parents are exchanging incubation duties [26,52].

It is possible that the higher daytime egg turning rate in Forster’s terns could also be a result of ‘dipping’, a behavior exhibited during the hottest part of the day [38]. This involves flying from the nest and dipping the beak, feet, and breast feathers into the water before quickly returning to the nest, which helps parents regulate the temperature of their eggs using evaporative cooling to prevent overheating [38,68]. Dipping has been observed in Forster’s terns nesting on the Salton Sea in southern California [38], but to our knowledge has not been reported at our study colonies in the San Francisco Bay.

Egg turning rate was not related to incubation stage in Forster’s terns. This was also observed in Adélie penguins [58,60**]** (but see [61]) as well as Laysan albatrosses and western gulls [55]. However, none of these species’ egg turning behavior has been studied over the complete duration of the incubation period. It is therefore not possible, based on these studies, to unequivocally determine whether egg turning rate changes over the full course of an incubation cycle in seabirds. Although several previous studies on terrestrial birds followed egg turning rate throughout the entire incubation period, the results were equivocal [69,70,71,72,73]. Therefore, more research is needed to fully address whether egg turning rates vary across the incubation period.

Hourly egg turning rate increased with nest initiation date. It seems plausible that less-experienced adults nest later in the summer than more-experienced adults, as occurs in common terns (*Sterna hirundo*) [59]. In contrast to our prediction that less-experienced adults would turn their eggs at a lower rate than more-experienced adults, it is also possible that less-experienced adults may instead turn their eggs at a higher rate. This relationship has not yet been investigated in any bird. The nest density of conspecifics at our colonies also increased over the course of the summer, and this may have amplified the level of disturbance between neighboring pairs, which may have caused parents to turn their eggs more frequently.

### Changes in egg orientation

Similar to other species [52], we found that the axis of orientation showing the greatest change variation during egg turning was about the yaw attitude, followed by roll, then pitch. Thus, when a bird sits down on the nest, it shifts its eggs so that they match the arrangement of its brood patches. Rotating the egg along the yaw axis is the easiest method of doing so, as observed in herring gulls [35] and black-headed gulls [52]. Conversely, in species with large clutch sizes and a single large brood patch, such as waterfowl, incubating adults do not need to manipulate each egg to individually fit into a brood patch. Rather, eggs are regularly rotated between the center and the periphery of the nest in order to maintain adequate egg temperatures [74].

Overall mean angle-change per egg turning event in Forster’s terns was 46.3 ± 0.5 deg. At a rate of 3.8 turns h^-1^, this equates to a mean angle-change of 176 ± 1.8 deg h^-1^, which is greater than that of any species studied to date [5]. Interestingly, 45 deg per egg-turn has been found to be the optimal turning angle for poultry [75,76], and is the standard angle change for artificial incubators [77]. However, artificial incubators typically operate at just 1 turn per hour [78].

### Egg temperature in Forster’s terns

Mean egg temperature in Forster’s terns was 40.0 ± 2.5 °C during the day and 38.9 ± 2.4 °C at night. This is higher than what has been found for other *Sterna* terns, whose egg temperatures typically range between 35 °C and 38 °C [56,79,80]. One reason for this could relate to interspecific differences in brood patch temperature; unfortunately, brood patch temperatures have not been reported for any *Sterna* species. Another potential reason is that we used artificial eggs that approximated, but did not precisely replicate, the density of genuine eggs.

Day vs. night was a significant predictor of egg temperature in Forster’s terns, which were on average 1.0 °C higher during the day than at night (Fig 2B). A likely explanation for this fluctuation in egg temperature is the daytime increase in ambient temperatures, which has been associated with increased egg temperature in Forster’s terns nesting at the Salton Sea [56].

Forster’s terns egg temperatures were more variable during day than at night. This difference in variability is likely due to the behavior of the parents, which often leave the nest unattended for short periods during the day. At night, both parents are often present [37]. The eggs of western gulls also exhibit increased temperature variability during the day, likely because this species is more active during the day [52,64]. Seabirds that exhibit a lower frequency of turnover between adults during incubation (e.g. albatrosses), or that nest in cavities (e.g. auklets), do not exhibit such profound differences in egg temperature variability between day and night [26,52,54,58,60].

Hourly egg temperature related positively to standardized nest initiation date, which may be related to environmental temperature. Nest initiation date ranged from 13 May to 9 July, and mean air temperature increased over the course of this time period. Another possible explanation is that less experienced adults tend to breed later in the summer, and may be less adept than more experienced adults at keeping their eggs adequately cool.

**Fig 3:**
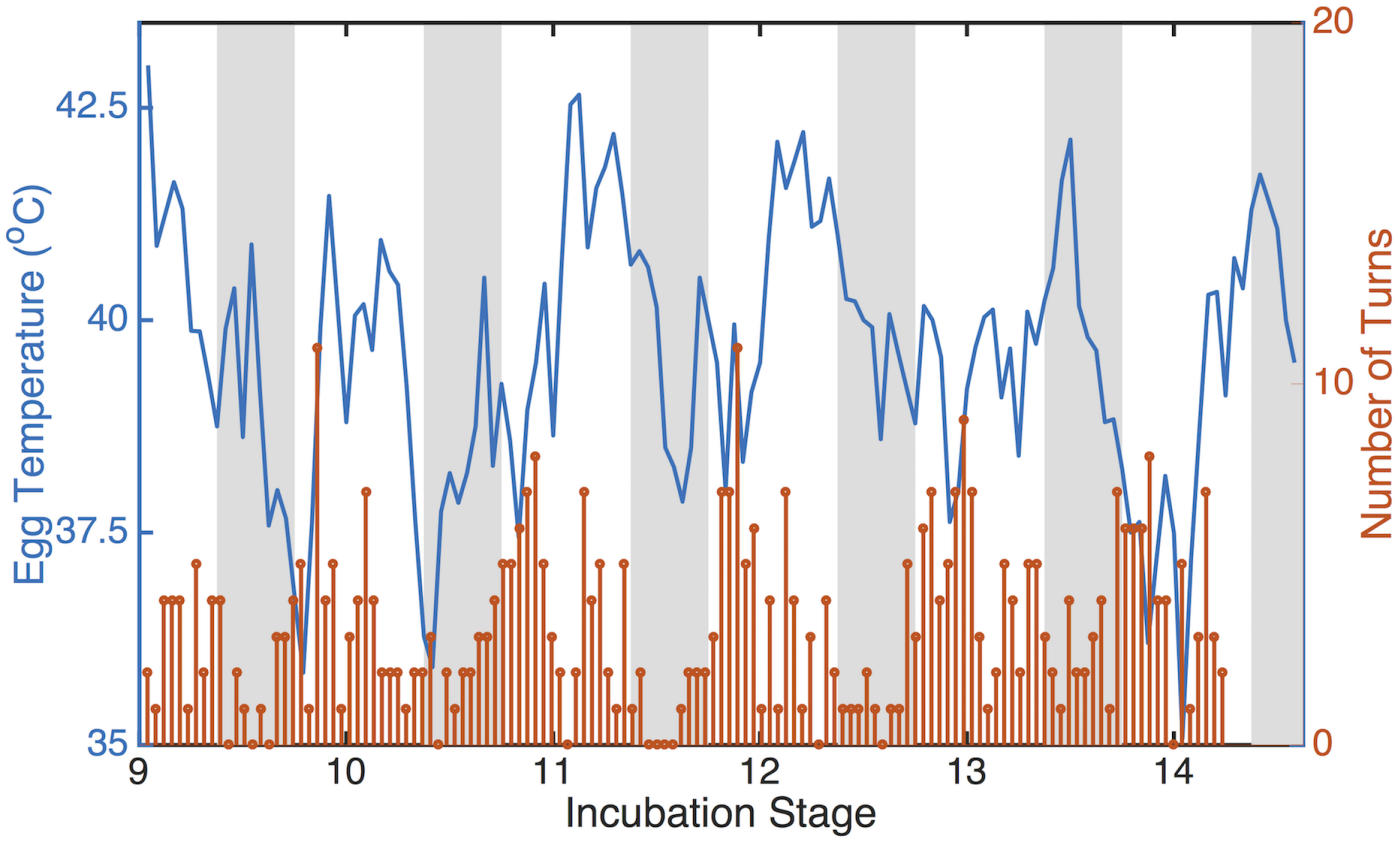
Five days of egg temperature and egg turn rate from a single tern nest. The blue line represents egg temperature, and the orange bars represent the number of egg turns in each hour. White areas indicate daytime, while gray areas indicate nighttime.

Hourly egg temperature was positively related to hourly egg turning rate (Fig 3). This relationship is seen in Cassin’s auklets [26], though egg temperature is negatively correlated with turning rate at night, when the parents are exchanging incubation duties. These auklets show no relationship between egg temperature and egg turning rate during the day. In Forster’s terns, the daytime exchange of incubation duties exposes the eggs to direct sunlight (and heat), possibly increasing egg temperature. These two examples suggest that the timing of parental exchange of incubation duties (and thus egg turning rate) may be associated with fluctuations in egg temperature. The small egg size of both species—approximately 27.5 g in Cassin’s auklets [81] and 21.3 g in Forster’s terns [66]—likely plays a role in facilitating the effect of egg turning on egg temperature. Small eggs have a low thermal inertia, and thus will heat and cool more quickly than large eggs. In contrast, western gulls and Laysan albatrosses exhibit no relationship between egg temperature and egg turning rate [52]. This could be due to a lack of diurnal fluctuations in the parental exchange of nest incubation duties in these species.

### Effects of mercury on incubation behavior

Suboptimal egg turning rates [1,4,14,27] and abnormal egg temperatures [13,15,28,29] have been shown to cause embryo malpositioning and other impairments that can reduce egg hatchability, and therefore were plausible pathways by which mercury could impair egg hatching success. However, despite the high variability in egg turning rate (3.8 ± 0.8 turns h^-1^), egg temperature (daytime: 40.0 ± 2.5 °C, nighttime: 38.9 ± 2.4 °C) and egg THg concentrations between nests (1.47 ± 0.76 μg/g fww), and the findings that mercury levels in this population are sufficiently high to reduce egg hatchability [12], egg turning rate or egg temperature were not correlated with THg concentrations in surrogate eggs from the same nest.

We deployed artificial eggs with accelerometers at 6–12 days in incubation, rather than during egg laying, to reduce the potential for nest abandonment. However, it is possible that egg turning behavior is more likely to be affected by mercury contamination of the parents during early incubation. Another possibility is that, because males (3.86 ± 0.51 μg/g wet wt) exhibit higher blood mercury concentrations than females (2.23 ± 0.30 μg/g wet wt; [49]), mercury may only alter incubation behavior in males. Unfortunately, we were unable to discriminate between male and female incubation behavior in our analyses. It is also plausible that, instead of lowering hatching success by altering parental behavior, mercury acts directly on the embryo, impairing neurological development and thereby inhibiting the ability of the chick to properly orient itself in preparation for hatching [12]. It is also possible that mercury may exhibit a dose response, in which a relationship between mercury contamination and incubation behavior is only detected above a certain mercury concentration and therefore any effects may not have been detectable in our dataset where sample size decreased with higher egg mercury concentrations.

### Application

Our results for egg turning behavior and egg temperature offer important insights that are relevant to avian conservation. More than 200 threatened bird species might benefit from artificial incubation [82]. Artificial incubation plays a particularly important role in the recovery of the critically endangered New Zealand fairy tern (*Sternula nereis davisae*), a close relative of the Forster’s tern, whose population has been severely impacted by introduced species [83]. Our findings on the egg turning rate, egg angle, and egg temperature of Forster’s terns suggest a larger amount of variation in egg turning behavior among seabirds than was previously expected. This underscores the importance of studying incubation behavior in a wide range of species in order to maximize the success of artificial incubation. Whether artificial incubation is effective for a particular species is largely determined by the egg turning rate and temperature of the incubator, two critical determinants of hatchability [5,6,84,85]. The standard egg turning rate in an artificial incubator is 1 turn per hour, with a 45-degree rotation about the long axis [6,78]. The amount of change in the pitch (89.1 ± 1.1 degrees h^-1^) that we observed in Forster’s terns was particularly surprising, though whether it is a functional aspect of egg turning remains unclear. Artificial incubators generally do not control changes in pitch, nor are such manipulations involved in poultry production [86]. Our findings also indicate that taxonomic order is not always a useful predictor of egg turning rate, as can be seen in the different egg turning rates of western gulls and Forster’s terns, both from the Order Charadriiformes. Instead, albumen content of the egg may be a better predictor of egg turning rate, as suggested by Deeming [5]. In addition to egg turning rate, our findings on egg temperature show that the typical artificial incubator may not be suitable for some species. Incubators typically operate at 37–38 °C, which has been found to be the optimum for galliform eggs [87], but which largely falls below the egg temperatures we recorded for Forster’s terns and other species [52]. Our results show that studying the natural incubation behavior of wild birds may have a role to play in increasing the effectiveness of conservation measures such as artificial incubation.
